# Association Between Serum Glycated Hemoglobin Levels at Early Gestation and the Risk of Subsequent Pregnancy Loss in Pregnant Women Without Diabetes Mellitus: Prospective Cohort Study

**DOI:** 10.2196/46986

**Published:** 2023-12-12

**Authors:** Xiaotian Chen, Yi Zhang, Hongyan Chen, Yalan Dou, Yin Wang, Wennan He, Xiaojing Ma, Wei Sheng, Weili Yan, Guoying Huang

**Affiliations:** 1 Department of Clinical Epidemiology & Clinical Trial Unit Children’s Hospital of Fudan University National Children’s Medical Center Shanghai China; 2 Pediatric Heart Center Children’s Hospital of Fudan University National Children’s Medical Center Shanghai China; 3 Shanghai Key Laboratory of Birth Defects, Children’s Hospital of Fudan University Shanghai China; 4 Research Unit of Early Intervention of Genetically Related Childhood Cardiovascular Diseases (2018RU002) Chinese Academy of Medical Sciences Shanghai China

**Keywords:** glycated hemoglobin, hemoglobin A1c, spontaneous pregnancy loss, gynecology, gynecological, obstetric, obstetrics, prospective cohort study, cohort, risk, risks, miscarriage, miscarriages, adverse outcome, adverse outcomes, risk ratio, pregnant women, pregnancy loss, gestational diabetes, fetal death, glycemic control, women, diabetes, diabetic, HbA1c, gestational, maternal, fetus, fetal, HbA1c levels, metabolic health, pregnant, pregnancy, association, associations, associated

## Abstract

**Background:**

As a severe morbidity during pregnancy, the etiology of spontaneous pregnancy loss (SPL) remains largely unknown. Serum glycated hemoglobin (HbA_1c_) level is an established predictor of SPL risk among women with diabetes, but little is known about whether such an association exists among pregnant women without diabetes when glycemic levels are within the normal range.

**Objective:**

This study aimed to quantify the association between maternal HbA_1c_ levels in early pregnancy and subsequent SPL risk in a cohort of pregnant women without diabetes.

**Methods:**

This prospective cohort study involved 10,773 pregnant women without diabetes enrolled at their first antenatal care visit at a hospital’s early pregnancy clinic from March 2016 to December 2018 in Shanghai, China. HbA_1c_ and fasting blood glucose (FBG) levels were examined at enrollment. Participants with diabetes before or pregnancy or those diagnosed with gestational diabetes were excluded. Diagnosis of SPL, defined as fetal death occurring before 28 gestational weeks, was derived from medical records and confirmed via telephone interviews. We used generalized linear models to quantify the associations of continuous and dichotomized maternal HbA_1c_ levels with SPL risk and reported crude and adjusted risk ratios (RRs) and 95% CIs. A restricted cubic spline (RCS) regression model was used to assess the potential nonlinear dose-response relationship. Adjusted covariates included maternal age, education level, preconception BMI, gestational weeks, gravidity, history of adverse pregnancy outcomes, family history of diabetes, folic acid supplementation, and smoking and drinking during the periconception period.

**Results:**

In total, 273 (2.5%) SPL cases occurred. Every 0.5% increase in HbA_1c_ levels was linearly associated with a 23% increase in SPL risk (adjusted RR [aRR] 1.23; 95% CI 1.01-1.50). The RCS model revealed that this association was linear (*P*=.77 for the nonlinearity test). Analyses based on dichotomized HbA_1c_ levels showed a significantly increased risk of SPL when HbA_1c_ levels were ≥5.9% (aRR 1.67; 95% CI 0.67-3.67), and the significance threshold was ≥5.6% (aRR 1.60; 95% CI 1.01-2.54). Sensitivity analyses showed similar results when including the participants with missing SPL records or HbA_1c_ data. Linear associations of HbA_1c_ levels remained significant even in the subgroups without overweight, alcohol consumption, and a family history of diabetes and adverse pregnancy outcomes. Every 1 mmol/L increment in maternal FBG levels was associated with a >2-fold higher risk of SPL (aRR 2.12; 95% CI 1.61-2.80; *P*<.001).

**Conclusions:**

Higher HbA_1c_ levels in early pregnant women without diabetes are associated with an increased SPL risk in a dose-response manner. Pregnant women with an HbA_1c_ level above 5.6% at early gestation need attention for its potentially increased risk for SPL. Our findings support the need to monitor HbA_1c_ levels to identify individuals at high risk of subsequent SPL in the general population of pregnant women.

**Trial Registration:**

ClinicalTrials.gov NCT02737644; https://clinicaltrials.gov/study/NCT02737644

## Introduction

Spontaneous pregnancy loss (SPL), also known as spontaneous abortion, is one of the serious morbidities during pregnancy and precedes an increased risk of reduced fertility, long-term depression, and anxiety among pregnant women [1]. As the definition of SPL varies between countries and international organizations, estimates of the prevalence of SPL vary among previous studies [2]. Data from a large nationally representative survey from the United States showed that approximately 20% of clinically recognized pregnancies ended in SPL (including stillbirths and ectopic or tubal pregnancies) during the whole gestation period [3]. Pregnancy losses from the first antenatal care visit to that at 28 weeks’ gestation are recorded in practice in China [4,5]. A nationwide study with 6.4 million medical records of Chinese pregnant women reported 2.8% of pregnancy losses before 28 weeks of gestation [6]. To date, the mechanism underlying the etiology of SPL remains largely unknown, and over 50% of women with SPL have no identified risk factors [7,8].

Poor glycemic control during pregnancy is an established independent predictor for adverse pregnancy outcomes [9]. Serum glycated hemoglobin (HbA_1c_) levels are conventionally used for monitoring blood glucose control [10]. According to the American Diabetes Association’s (ADA’s) clinical practice guidelines, individuals with HbA_1c_ levels within 5.7%-6.4% and ≥6.5% are classified as having prediabetes and diabetes, respectively, in the general population, and HbA_1c_ levels of 5.9% and higher in pregnant women are considered an early indicator of abnormal glucose metabolism and a higher risk of adverse pregnancy and neonatal outcomes [11]. Previous studies have reported associations of HbA_1c_ levels of pregnant women with insulin-dependent diabetes and SPL risk [12-14]. However, little is known about these associations in pregnant women without diabetes.

An HbA_1c_ test at the first prenatal visit has been recommended for those at risk of developing gestational diabetes only, including having obesity and having a family history of gestational diabetes, but not yet for the general population of pregnant women [11,15,16]. In this prospective cohort study, we aimed to evaluate whether HbA_1c_ levels in pregnant women without diabetes are associated with a subsequent SPL risk.

## Methods

### Study Population

Pregnant women included in this study were a subcohort of the ongoing Shanghai Preconception Cohort Study (SPCC; ClinicalTrials.gov NCT02737644) [17], who were enrolled between March 2016 and December 2018 from one of the study sites—a tertiary maternity hospital where HbA_1c_ and fasting blood glucose (FBG) levels were routinely examined at the first antenatal visit for all pregnant women. This maternity hospital is one of the largest delivery hospitals (with >20,000 births per year) during the study period and accounts for over 20% of the annual deliveries in the city. Participants were enrolled at their first antenatal visit at early pregnancy clinics, and each woman had only one medical record for this study. Biochemistry and SPL diagnosis data were extracted from the hospital’s electronic medical record system. Among all pregnant women during the study period (N=13,129), 10,773 were eligible for the primary analysis after the exclusion of those who met any one of the following criteria: missing medical records after the first antenatal visit, missing information regarding HbA_1c_ or FBG levels at entry, having received artificial abortions, self-reported diabetes before pregnancy (ie, an HbA_1c_ level of ≥6.5% or FBG level of ≥7.0 mmol/L at the first antenatal visit), or having received insulin treatment during pregnancy or taking oral hypoglycemic drugs before or during pregnancy. We defined this group as pregnant women without diabetes.

### Ethical Considerations

Ethics approval for this study was sought from the institutional Ethics Committee of the Children’s Hospital of Fudan University (2016–49). Written informed consent was obtained from all participants before recruitment. All data were anonymously analyzed.

### Exposures and Covariates

We treated the fasting HbA_1c_ levels in early pregnancy as the main exposure in this study. Given that FBG levels are also an index of glycemic control, both HbA_1c_ and FBG levels were abstracted from the medical records for analyses. According to the routine practice of the hospital where this subcohort was recruited, HbA_1c_ and FBG levels were measured in 2 hours using a venous blood sample after overnight fasting (>8 hours of fasting) at their first antenatal care visit. Venous blood for the HbA_1c_ test was collected in an EDTA-containing tube and determined using high-performance liquid chromatography (Bio-Rad) in the hospital’s certified standard clinical examination center following standard protocols.

A series of variables regarding known or suspected risk factors for SPL were considered covariates in the association analysis [18]. As described elsewhere, demographic characteristics and pregnancy history of the participants upon enrollment were collected through a prespecified standard self-administered questionnaire and an interview with the obstetric nurse during the first antenatal visit. Maternal preconception BMI (pre-BMI) was calculated using self-reported measures of prepregnancy body weight and categorized as normal weight or overweight. We used both the Chinese standard (≥24 kg/m^2^) and the international standard (≥25 kg/m^2^) to define the overweight status [19,20]. We defined a family history of diabetes as having at least 1 first-degree relative diagnosed with diabetes. Smoking exposure and alcohol drinking were defined as smoking cigarettes or having been exposed to second-hand cigarette smoke, and as consuming any alcoholic beverages within 3 months before or during the current pregnancy, respectively. Folic acid supplementation (FAS) was defined as having a regular intake of pure folic acid tablets or multivitamins containing folic acid before or during early pregnancy. Gestational weeks at enrollment were routinely determined by the last menstruation period and confirmed through a routine ultrasonographic examination. Participants were defined as having a history of adverse pregnancy outcomes if they had abortions, preterm delivery, stillbirths, or ectopic pregnancy in previous pregnancies.

### Outcomes

The recorded SPL cases from the hospital’s electronic medical system were fetal deaths occurring before 28 gestational weeks in accordance with the Chinese clinical guidelines [4]. Stillbirth (fetal deaths after 28 gestational weeks), and artificial abortions due to ectopic pregnancies, molar pregnancies, or any clinically recognized disorders were not considered SPL in this study. Trained staff verified these diagnoses through a personal telephone interview with pregnant women or their husbands before the analysis.

### Statistical Analysis

Continuous variables were reported as mean (SD) values for a normal or approximate normal distribution and median (IQR) values for a skewed distribution, and 2-tailed unpaired Student *t* tests or Mann-Whitney *U* tests were used for comparisons between the SPL and non-SPL group, respectively. Normality was visually inspected using frequency histograms. Categorical variables were summarized as frequencies and percentages, and chi-square tests were used for the group comparisons.

Our primary aim was to investigate the associations of HbA_1c_ and FBG levels as continuous variables with SPL risk. We used generalized linear models with binomial family and log link functions treating HbA_1c_ (rescaled through dividing by 0.5) and FBG levels as continuous variables to estimate crude and adjusted risk ratios (aRRs) and 95% CIs. A 0.5% absolute increment was chosen for the HbA_1c_ level because it reflects a clinically important change [21]. Considering that a HbA_1c_ level of 5.9% in early pregnancy has been suggested as the cutoff for identifying women at increased risk of adverse pregnancy outcomes [11], we further assessed the associations based on dichotomous HbA_1c_ (coded as 1 for a HbA_1c_ level of ≥5.9% and 0 for an HbA_1c_ level of <5.9%). We used a restricted cubic spline (RCS) regression model with 3 knots (5th, 50th, and 95th percentile levels) fitted in R (*rms* package) to assess the potential nonlinear dose-response relationship of HbA_1c_ levels with SPL risk. Adjusted covariates included maternal age, education level, pre-BMI, gestational week at HbA_1c_ examination, gravidity, history of adverse pregnancy outcomes, family history of diabetes, FAS, and exposure to smoking and drinking during the periconception period.

We conducted an exploratory analysis to investigate the HbA_1c_ cutoff indicating pregnant women at an increased risk of SPL by using a series of regression models using HbA_1c_ levels from the median level of the study population (5.1%) to 6.0% in a 0.1% interval without adjustment for multiple testing.

As there were 2147 participants—1360 (10.5%) with missing medical records after the first antenatal visit and 787 (6.1%) with missing HbA_1c_ data—we conducted 2 sensitivity analyses to test the robustness of the main results after multiple imputation with chained equations based on a missing at random assumption. First, we added 1360 pregnant women with missing SPL data after imputation (sensitivity analysis 1: n=10,773+1360). Second, we included 787 pregnant women with missing HbA_1c_ levels at enrollment after imputation (sensitivity analysis 2: n=10,773+787). To ensure that our main results are free from potential bias from unmeasured confounders, we further repeated the primary association analyses within subgroups with a potentially low risk of SPL, including pregnant women younger than 35 years, without overweight, a family history of diabetes, a history of adverse pregnancy outcomes, or smoking or alcohol drinking exposures. Statistical analyses were performed by Stata (version 16.0; StataCorp) and R package (version 3.6.1; The R Foundation), and all statistical tests were 2-sided at a significance level of .05. A post hoc power analysis was performed for the main association analyses of continuous HbA_1c_ levels with SPL risk, which revealed that with the current sample size, associations with a risk ratio (RR) lower than 0.84 or greater than 1.19 for continuous exposures of interest will ensure a power of ≥80% at an α level of 5%.

## Results

### Characteristics of the Study Population

**Figure 1:**
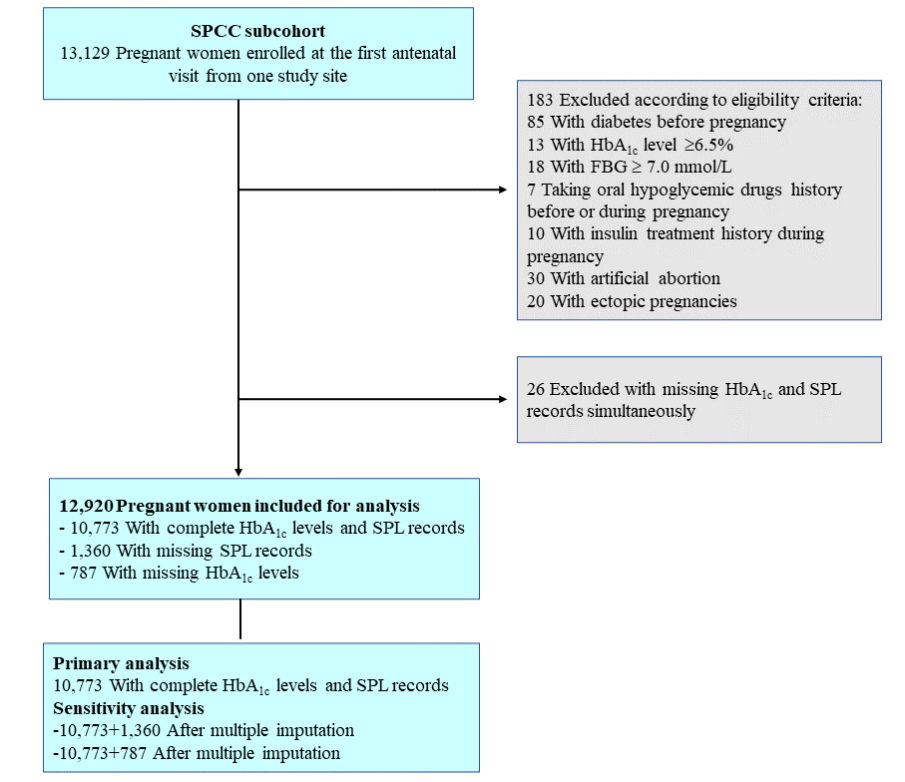
Flowchart of the study population. HbA_1c_: serum glycated hemoglobin; SPCC: Shanghai Preconception Cohort Study; SPL: spontaneous pregnancy loss.

A total of 10,773 eligible pregnant women were included in the main analysis (Figure 1), with a mean age of 30.5 (SD 4.0) years and an average of 10.8 (SD 1.7) weeks of gestation at enrollment (Table 1). The majority of participants (n=9866, 91.6%) had a college or higher level of education, 4394 (40.9%) had more than 1 gravidity, 2056 (19.1%) had overweight before pregnancy, and 3874 (36.3%) had a family history of diabetes. Smoking and alcohol consumption were reported by 1043 (9.8%) and 857 (8.0%) pregnant women, respectively. Besides, 2817 (26.4%) pregnant women had a history of adverse pregnancy outcomes. The distribution of HbA_1c_ and FBG levels approximately met a normal distribution, with a mean value of 5.09% (SD 0.30%) and 4.49 (SD 0.78) mmol/L, respectively. The 2 markers were weakly correlated (*r*=.19; *P*<.001) and overlapped in quartiles (Figure S1 in Multimedia Appendix 1). We summarized the characteristics of the 10,773 eligible pregnant women and 2147 women with missing medical records or HbA_1c_ data (Table S1 in Multimedia Appendix 1), which were overall similar.

**Table 1:** Baseline characteristics of study population at the first antenatal care visit.

Variables		Total sample (N=10,773)	SPL^a^ group (n=273)	Non-SPL group (n=10,500)	*P* value
Age^b^ (years), mean (SD)		30.5 (4.0)	32.0 (4.4)	30.4 (3.9)	<.001
Pre-BMI (kg/m^2^), mean (SD)		21.7 (2.9)	22.5 (3.0)	21.7 (2.9)	<.001
**Pre-BMI categories, n (%)**					
	≥24 kg/m^2^	2056 (19.1)	65 (23.8)	1991 (19.0)	.047
	≥25 kg/m^2^	1347 (12.5)	47 (17.2)	1300 (12.4)	.02
	Missing	32 (0.3)	—^c^	32 (0.3)	N/A^d^
Gestational weeks at enrollment^e^, mean (SD)		10.8 (1.7)	10.7 (1.6)	10.7 (1.7)	.48
**Education level, n (%)**					.24
	College and above	9866 (91.6)	247 (90.5)	9619 (92.4)	
	Below college	817 (7.6)	26 (9.5)	791 (7.6)	
	Missing	90 (0.8)	—	90 (0.8)	
**Gravidity, n (%)**					.26
	1	6357 (59.1)	149 (54.6)	6208 (59.2)	
	2	2759 (25.7)	75 (27.5)	2684 (25.6)	
	≥3	1635 (15.2)	49 (18.9)	1586 (15.1)	
	Missing	22 (0.2)	2 (0.7)	20 (0.1)	
**History of adverse pregnancy outcomes, n (%)**		2817 (26.4)	93 (34.1)	2724 (26.2)	.003
	Missing	93 (0.9)	1 (0.4)	92 (0.9)	N/A
**Family history of diabetes, n (%)**		3874 (36.3)	112 (41.0)	3762 (36.2)	.10
	Missing	104 (1.0)	5 (1.8)	99 (0.9)	N/A
**FAS^f^ before or during early pregnancy, n (%)**		8022 (75.1)	240 (87.9)	7804 (75.0)	.06
	Missing	90 (0.8)	4 (1.5)	86 (0.8)	N/A
**Smoking exposure, n (%)**		1043 (9.8)	33 (12.1)	1010 (9.7)	.19
	Missing	91 (0.8)	2 (0.7)	89 (0.8)	N/A
**Alcohol drinking, n (%)**		857 (8.0)	19 (7.0)	838 (8.1)	.51
	Missing	91 (0.8)	3 (1.1)	88 (0.8)	N/A
FBG^g,h^ (mmol/L), mean (SD)		4.49 (0.78)	4.64 (0.39)	4.48 (0.47)	<.001
FBG≥6.1 mmol/L, n (%)		29 (.3)	1 (.4)	28 (.3)	.75
HbA_1c_^i^ (%), mean (SD)		5.09 (0.30)	5.15 (0.33)	5.09 (0.30)	.002
HbA_1c_≥5.9%, n (%)		126 (1.2)	6 (2.2)	120 (1.1)	.03

^a^SPL: spontaneous pregnancy loss.

^b^Age data were missing for 23 out of 10,773 (0.2%), 3 out of 273 (1.1%), and 20 out of 10,500 (0.2%) pregnant women in the total sample, SPL group, and non-SPL group, respectively.

^c^Not available.

^d^N/A: not applicable.

^e^Data for gestational weeks at enrollment were missing for 60 out of 10,773 (0.6%), 2 out of 273 (0.7%), and 58 out of 10,500 (0.5%) pregnant women in the total sample, SPL group, and non-SPL group, respectively.

^f^FAS: folic acid supplementation.

^g^FBG: fasting blood glucose.

^h^FBG levels were ≥6.1% for 29 out of 10,773 (0.3%), 1 out of 273 (0.4%), and 28 out of 10,500 (0.3%) pregnant women in the total sample, SPL group, and non-SPL group, respectively (*P*=.75).

^i^HbA_1c_: serum glycated hemoglobin.

The incidence rate of SPL was 2.5% among 10,773 pregnant women in this study. Compared to pregnant women without SPL, those with SPL were 1.6 years older (mean ages of the SPL and non-SPL groups were 32.0, SD 4.4 vs 30.4, SD 3.9 years, respectively) during pregnancy, were more likely to have overweight before pregnancy (23.8% vs 19.0%), and had higher levels of HbA_1c_ (5.15% SD 0.33% vs 5.09% SD 0.30%; albeit in the clinically normal range) and FBG (4.64, SD 0.39 vs 4.48, SD 0.47 mmol/L) at the first antenatal visit.

### Associations Between Maternal HbA1c and FBG Levels and SPL Risk

**Figure 2:**
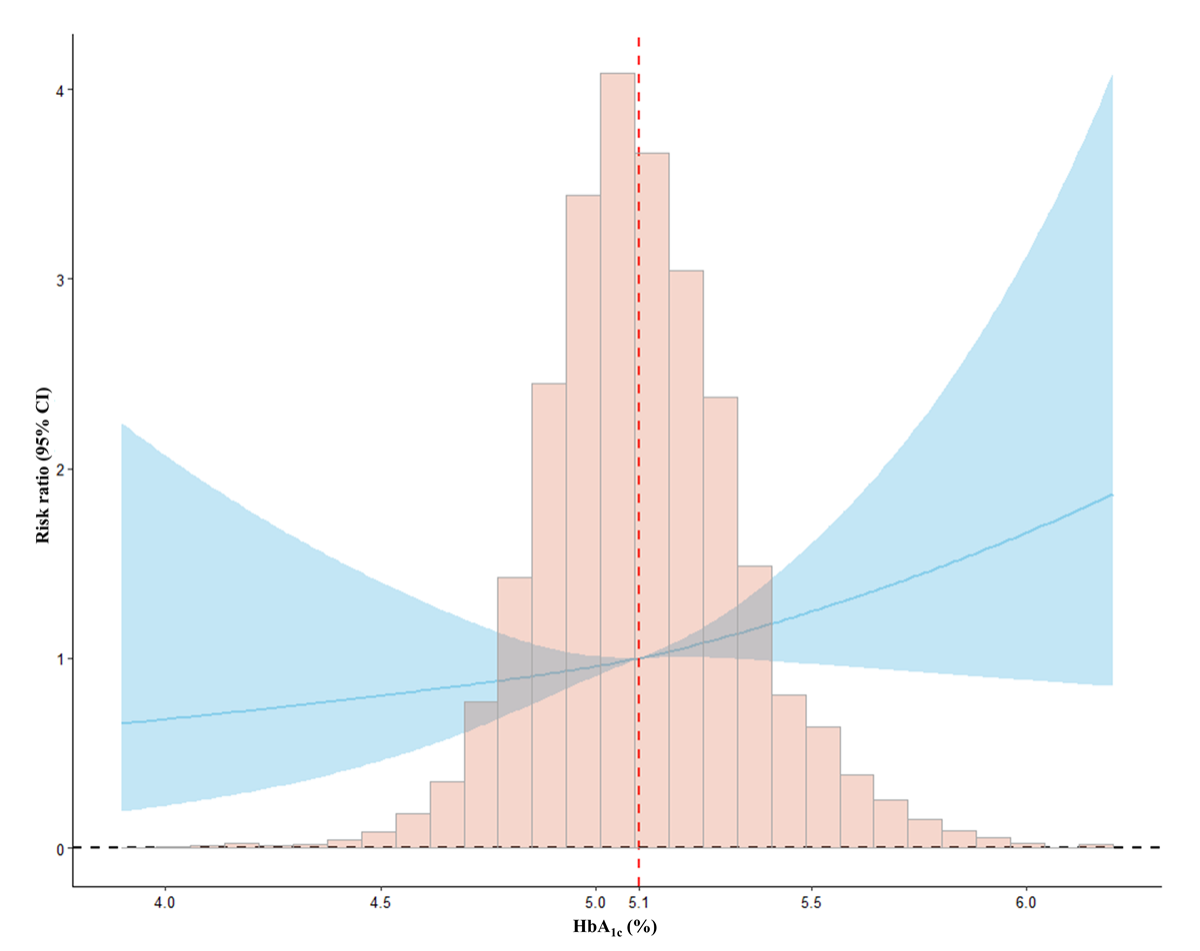
Restricted cubic spline plots for the association between maternal serum glycated hemoglobin (HbA_1c_) levels in early pregnancy with spontaneous pregnancy loss (SPL) risk.

As shown in Table 2, maternal HbA_1c_ levels showed significant positive associations with SPL risk with an unadjusted RR of 1.34 (95% CI 1.10-1.63) per 0.5% increase in HbA_1c_ levels, and the association remained significant after adjusting for covariates (aRR 1.23, 95% CI 1.01-1.50; *P*=.04). The RCS model showed a linear association of SPL risk with increasing HbA_1c_ levels through the whole range of HbA_1c_ values (*P*=.77 for the nonlinearity test; Figure 2). No significant association was found between a HbA_1c_ level of ≥5.9% and SPL risk after adjusting for covariates. Every 1 mmol/L increment in maternal FBG levels was associated with a >2-fold higher risk of SPL (aRR 2.12, 95% CI 1.61-2.80; *P*<.001).

**Table 2:** Associations between maternal HbA_1c_^a^ and FBG^b^ levels and SPL^c^ risk during early pregnancy.

Analysis			SPL group/total sample, n/n	Crude model		Adjusted model^d^	
				Risk ratio (95% CI)	*P* value	Risk ratio (95% CI)	*P* value
**Primary analysis**							
	**HbA_1c_ (per 0.5% increment)**		273/10,773	1.34 (1.10-1.63)	.004	1.23 (1.01-1.50)	.04
		<5.9%	267/10,647	Reference	N/A^e^	Reference	N/A
		≥5.9%	6/126	1.94 (.85-4.45)	.12	1.67 (0.76-3.67)	.20
	FBG (mmol/L)		273/10,773	2.38 (1.82-3.11)	<.001	2.12 (1.61-2.80)	<.001
**Sensitivity analysis 1 (Including 1360 pregnant women with missing medical diagnosis records based on imputation)**							
	**HbA_1c_ (per 0.5% increment)**		306/12,133	1.36 (1.11-1.67)	.003	1.25 (1.02-1.53)	.03
		<5.9%	299/11,993	Reference	N/A	Reference	N/A
		≥5.9%	7/140	2.02 (0.93-4.42)	.08	1.51 (0.68-3.34)	.31
**Sensitivity analysis 2 (including 787 pregnant women with missing HbA_1c_ levels based on imputation)**							
	**HbA_1c_ (per 0.5% increment)**		373/11,560	1.36 (1.10-1.69)	.005	1.25 (1.01-1.55)	.04
		<5.9%	366/11,430	Reference	N/A	Reference	N/A
		≥5.9%	7/130	1.59 (0.70-3.65)	.27	1.42 (0.49-2.67)	.75

^a^HbA_1c_: serum glycated hemoglobin.

^b^FBG: fasting blood glucose.

^c^SPL: spontaneous pregnancy loss.

^d^Adjusted for age, pre-BMI, gestational weeks, education, gravidity, history of abnormal pregnancy, family history of diabetes, folic acid supplementation, and drinking and smoking status.

^e^N/A: not applicable.

### Sensitivity and Subgroup Analyses

In the first sensitivity analysis including 1360 pregnant women with missing medical diagnosis records, the association between HbA_1c_ levels and SPL risk did not substantially change (aRR per 0.5% increment 1.25, 95% CI 1.02-1.53; *P*=.03; Table 2). Similar results were also observed in the second sensitivity analysis upon including pregnant women with missing data on HbA_1c_ levels (aRR per 0.5% increment 1.25, 95% CI 1.01-1.55; *P*=.04).

Our exploratory analyses showed that the strength of the associations increased markedly from below 1.2 to 1.6 at an HbA_1c_ level of 5.6% (aRR 1.60; 95% CI 1.01-2.54; *P*=.048) and increased further at higher cutoff levels, although significance was not achieved at HbA_1c_ levels of 5.9% and 6.0% (Figure S2 in Multimedia Appendix 1). In further subgroup analyses including low-SPL risk populations without overweight, alcohol drinking, a family history of diabetes, and a history of adverse pregnancy outcomes, the associations of every 0.5% increase in HbA_1c_ levels with SPL risk remained very similar compared to those in the main analysis (aRR 1.32, 95% CI 1.07-1.63; aRR 1.18, 95% CI 1.01-1.38; aRR 1.24, 95% CI 1.03-1.48; and aRR 1.73, 95% CI 1.26-2.36; respectively; Table S2 in Multimedia Appendix 1).

## Discussion

### Principal Findings

In this large prospective cohort study, we provide solid evidence that among pregnant women without diabetes, HbA_1c_ levels in early gestation within the clinically normal range were associated with an increased risk of SPL in a linear dose-response manner. Although far below the recommended threshold for diagnosing gestational diabetes, HbA_1c_ levels may indicate an increased subsequent SPL risk in pregnant women in general. Our findings in a prospective cohort are novel and deepen our understanding of the important pathophysiologic role of impaired maternal glycemic metabolism in the development of SPL.

### Comparison With Prior Work

Several studies have investigated the adverse effect of elevated HbA_1c_ levels on SPL risk only among pregnant women with diabetes and have reported conflicting findings [12-14,22]. A case-control study including 432 control women and 386 women with type 1 diabetes found that the rate of SPL did not significantly differ between the 2 groups (16.1% vs 16.2%, respectively) [12]. However, another case-control study among women with type 1 diabetes reported that elevated HbA_1c_ levels were associated with SPL risk [13]. Another cohort study of 573 women with type 1 diabetes observed a linear association between HbA_1c_ levels and the risk of adverse pregnancy outcomes including SPL when HbA_1c_ levels were >7.0% [14]. This is the first prospective cohort study demonstrating the link between maternal HbA_1c_ levels in early pregnancy and SPL risk among pregnant women without diabetes. These associations were still significant in the population at a low risk of SPL. We also observed a highly overlapped distribution of FBG levels among HbA_1c_ categories, and consistent associations with SPL were also observed with regard to FBG levels. However, regarding their clinical application, FBG level is a less ideal marker than HbA_1c_ level because the former is less stable, requires a fasting state for examination, and has relatively greater intraindividual variability [23]. Our findings suggest that attention to glycemic control should not be limited to pregnant women with diabetes but should also include those with high HbA_1c_ levels within the clinical range during early gestation for the related increased risk of SPL.

### Research Implications

Our study addresses 2 important issues with important clinical and research implications. First, our findings expand on the literature on risk factors for SPL, an adverse pregnancy outcome whose modifiable determinants remain poorly understood. Second, we address a risk factor that is known to be modifiable through lifestyle and pharmacological interventions. Measurement of HbA_1c_ levels during pregnancy is conventionally used to monitor glycemic control in pregnant women with diabetes [24]; this marker is superior to FBG levels, mainly for its greater stability but lesser variability among individuals and its nonrequirement of fasting [23]. Given the special physiological status of pregnancy, the target HbA_1c_ level for ideal glycemic control remains inconclusive. In China, based on recommendations for the general population, an HbA_1c_ level less than 6.5% is recommended to indicate ideal glycemic control before conception [25]. An HbA_1c_ level of ≥5.9% is recommended by the ADA’s guidelines as an indicator for screening pregnant women with a higher risk of preeclampsia, macrosomia, shoulder dystocia, and perinatal death [11]. Our findings add new evidence for the linear relationship between glucose metabolism markers and SPL risk when HbA_1c_ levels are clinically normal [11], and the magnitudes of the associations are even stronger in those exposed to smoking or those who have a history of adverse pregnancy outcomes.

The mechanism underlying the association between maternal HbA_1c_ levels and SPL in women without diabetes remains unclear. Animal studies found that poor glycemic control may facilitate premature programmed cell death of key progenitor cells of the blastocyst and promote pregnancy loss [26]. In addition, poor glycemic control may interfere with implantation by inhibiting trophectoderm differentiation and increasing oxidative stress, thus affecting the expression of critical genes essential for embryogenesis [27,28]. Further population studies exploring the biological mechanisms underlying the association between HbA_1c_ levels and SPL in pregnant women without diabetes are warranted.

The findings of our exploratory analysis are clinically relevant, indicating that pregnant women with HbA_1c_ levels above 5.6% need attention for owing to a potentially increased risk of SPL. Given that HbA_1c_ levels are 0.5%-0.6% lower in early pregnancy than in nonpregnant women [29], HbA_1c_ levels over 5.6% at early gestation may reflect an elevated HbA_1c_ status that occurred before or at pregnancy (ie, prediabetes status), which accounts for over 13% of the population [6]. Another large cohort study and systematic review have reported associations between an HbA_1c_ level of <6.4% with increased severe maternal morbidity and subsequent gestational diabetes mellitus [30,31]. Taken together, based on the aforementioned evidence and our findings, we propose that monitoring of HbA_1c_ levels in early pregnancy is necessary general pregnant women, and HbA_1c_ levels exceeding 5.6% might be considered an indicator of high risk for SPL and additional medical care.

### Strengths and Limitations

The merits of our study include the prospective nature of the data, the large sample size, and the consistent results from robust analyses, which make our findings convincing. However, several limitations exist. First, residual confounding, such as uterine abnormalities, chromosomal abnormalities, antiphospholipid syndrome, and thyroid disorders, cannot be completely ruled out due to the nature of observational studies. Data on important biomarkers such as insulin levels during early gestation were not available. Second, in the exploratory analyses, we did not correct for multiplicity. Prospective multicenter cohort studies investigating whether a maternal HbA_1c_ level of 5.6% in early pregnancy could indicate an increased risk of SPL are warranted in the future. Third, compared with the rate of clinically registered pregnant women ending in SPL (11%-20%), the SPL rate in this cohort was much lower. Given the nature of the study population, which was recruited at their first antenatal care visit (with a median of 10 weeks of gestation), SPL occurred earlier than our observation was inevitably missed, selection bias exists, and our findings are not generalizable to the entire general population. The study population we selected, comprising pregnant women without diabetes, may include some cases of mild gestational diabetes diagnosed midgestation and without insulin treatment. Moreover, potential biases such as outcome misclassification could not be ruled out from the association analyses based on imputation.

### Conclusions

This study is the first to document that maternal HbA_1c_ levels in early pregnancy are associated with the subsequent risk of SPL in a dose-response manner among pregnant women without diabetes. Our findings support the need to monitor HbA_1c_ levels for identifying high risk of subsequent SPL in pregnant women in general and expand on the growing literature linking overall metabolic health to reproductive and pregnancy health among otherwise healthy women.

## Appendix

Multimedia Appendix 1 Additional figures, tables, and information.

## Abbreviations

ADA: American Diabetes Association
aRR: adjusted risk ratio
FAS: folic acid supplementation
FBG: fasting blood glucose
HbA_1c_: serum glycated hemoglobin
Pre-BMI: preconception BMI
RCS: restricted cubic spline
RR: risk ratio
SPCC: Shanghai Preconception Cohort Study
SPL: spontaneous pregnancy loss

## Data Availability

The data supporting the findings of this study are available from the corresponding author upon reasonable request.
