# Supplementary material for: Association Between Serum Glycated Hemoglobin Levels at Early Gestation and the Risk of Subsequent Pregnancy Loss in Pregnant Women Without Diabetes Mellitus: Prospective Cohort Study
Source: JMIR Public Health Surveill. 2023 Dec 12;9:e46986. doi: 10.2196/46986 (PMC10751628; doi:10.2196/46986)
Supplement: Multimedia Appendix 1 [file publichealth_v9i1e46986_app1.docx]

**Multimedia Appendix 1**

**Association Between Serum Glycated Hemoglobin Levels at Early Gestation and the Risk of Subsequent Pregnancy Loss in Pregnant Women Without Diabetes Mellitus: Prospective Cohort Study**

Xiaotian Chen, Msc^1^; Yi Zhang, MPH^1^; Hongyan Chen, PhD^1^; Yalan Dou, PhD ^1^; Yin Wang, PhD^1^; Wennan He, Msc^1^; Xiaojing Ma, MD^2,3^; Wei Sheng, PhD^3^; Weili Yan, PhD ^1,3,4^*; Guoying Huang, MD^2,3,4^*; On behalf of the SPCC group

**Affiliations:**

1. Department of Clinical Epidemiology & Clinical Trial Unit, Children’s Hospital of Fudan University, National Children’s Medical Center, Shanghai, China
2. Pediatric Heart Center, Children’s Hospital of Fudan University, National Children’s Medical Center, Shanghai, China
3. Shanghai Key Laboratory of Birth Defects, Shanghai, China

Research Unit of Early Intervention of Genetically Related Childhood Cardiovascular Diseases (2018RU002), Chinese Academy of Medical Sciences, Shanghai, China

**Contents**

**Figure S1**. The distribution of maternal HbA1c and fasting blood glucose levels in early pregnancy...................................................................................2

**Figure S2**. Risk ratios of SPL by different cut-off HbA1c levels..............................................................................................................................3

**Table S1**. Characteristics between pregnancies with or without complete medical records or HbA1c data .....................................................................................4

**Table S2**. Subgroup analysis for the associations of maternal HbA1c levels in early pregnancy with SPL risk.........................................................................5

**The Shanghai PreConception Cohort (SPCC) group**...............................................6


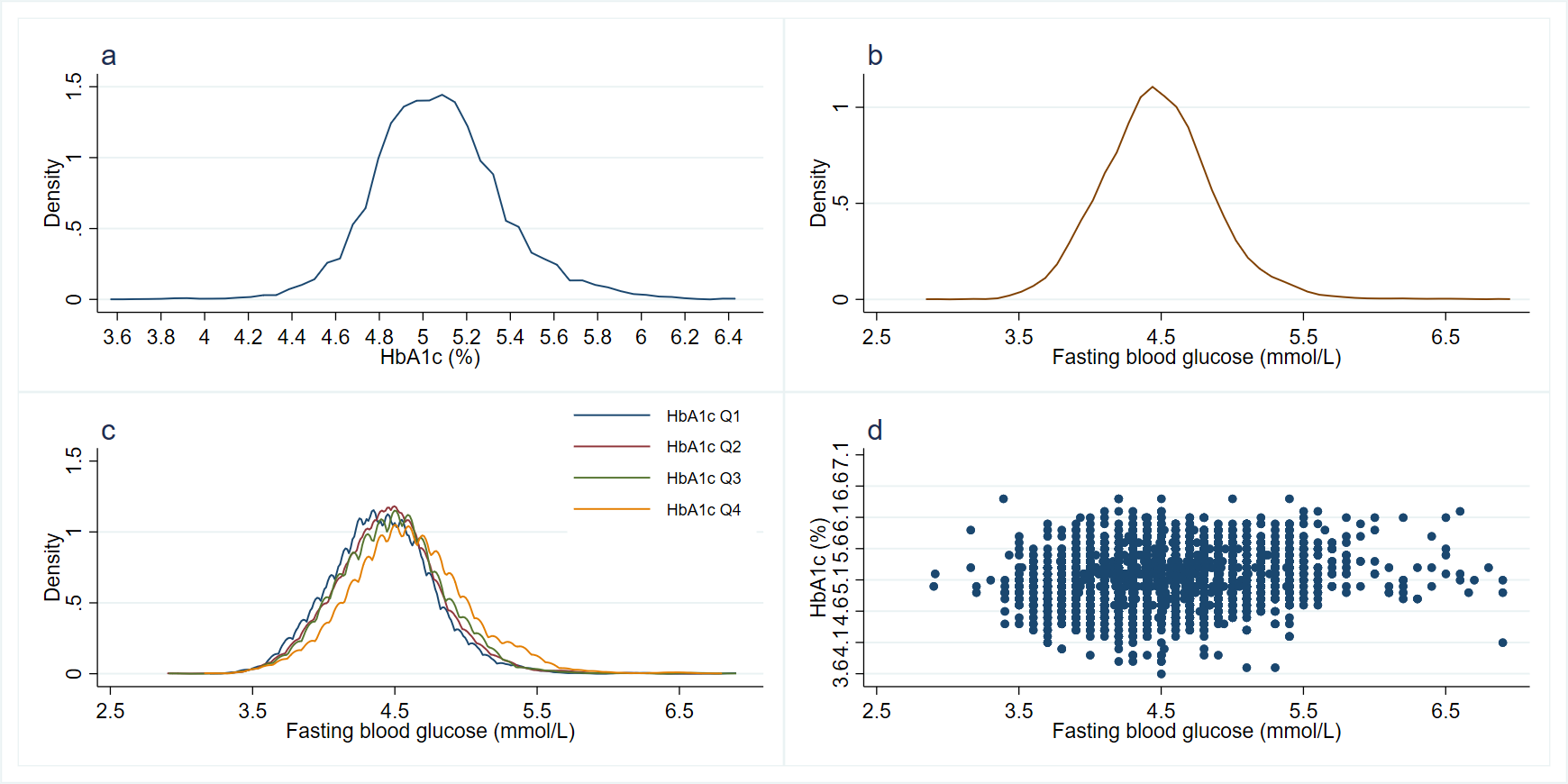


**Figure S1.** The distribution of maternal HbA1c and fasting blood glucose levels in early pregnancy. a-b, the kernel density plot of HbA1c and fasting blood glucose; c, the kernel density plot of fasting blood glucose according to HbA1c quartiles; d, the correlation of fasting blood glucose and HbA1c (*r*=.19, *P*<.001).


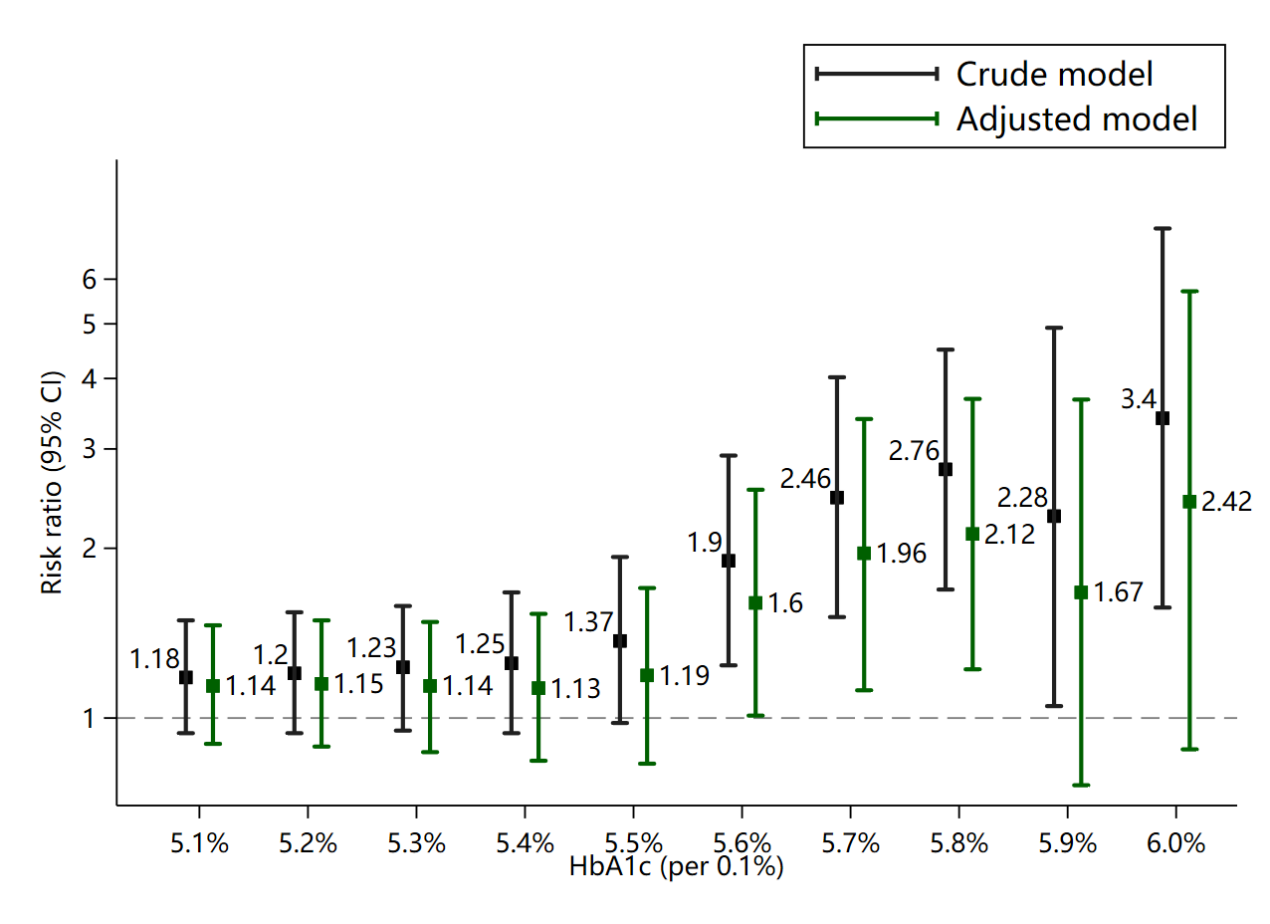


**Figure S2. Risk ratios of SPL by different cut-off HbA1c levels.** The statistically significant association occurred at HbA1c levels of 5.6%. Compared with pregnant women<5.6%, subjects with HbA1c ≥5.6% were associated with 60% increased risk of SPL (aRR, 1.60; 95% CI, 1.01-2.54; *P*=.048). The Y-axes was plotted on log scales. HbA1c, glycated hemoglobin A1c. The dashed line indicated risk ratio of 1.

**Table S1. Characteristics between pregnant women with or without** **complete medical records or HbA1c levels**

| **Variables** | **Pregnant women with complete medical records and HbA1c levels** | **Pregnant women with missing medical records or HbA1c levels** |
| --- | --- | --- |
| N | 10773 | 2147 |
| Age, mean±SD | 30.5±4.0 | 29.8±3.6 |
| Preconception BMI (kg/m^2^), mean±SD | 21.7±2.9 | 21.7±3.0 |
| Preconception Overweight, n (%) | 2056 (19.1) | 419 (19.5) |
| Gestational weeks at enrolment, mean±SD | 10.8±1.7 | 10.9±2.0 |
| Gravidity, n (%) |  |  |
| 1 | 6357 (59.1) | 1108 (51.6) |
| 2 | 2759 (25.7) | 576 (26.8) |
| ≥3 | 1635 (15.2) | 331 (21.6) |
| History of adverse pregnancy outcomes, n (%) | 2817 (26.4) | 588 (27.4) |
| Family history of diabetes, n (%) | 3874 (36.3) | 649 (30.2) |
| FAS before or during early pregnancy, n (%) | 8022 (75.1) | 1672 (77.9) |
| Smoking exposure, n (%) | 1043 (9.8) | 197 (8.9) |
| Alcohol drinking, n (%) | 857 (8.0) | 186 (8.6) |

Data were presented as mean±SD, and number (percentage). BMI, body mass index; IQR, inter-quartile range; FAS, folic acid supplementation; SD, standard deviation; SPL, spontaneous pregnancy loss. The characteristics of the subjects were not imputed in this table.

**Table S2. Subgroup analyses: associations of maternal** **HbA1c levels in early pregnancy with SPL** risk in low risk sub-populations

| **Subgroup** | **Adjusted RR (95% CI)** | ***P*** |
| --- | --- | --- |
| Pregnant women under 35 years (N=9130) | 1.38 (.95, 1.37) ^a^ | .17 |
| Pregnant women without overweight^*^ (BMI<24 kg/m^2^, N=8717) | 1.32 (1.07, 1.63) ^b^ | .008 |
| Pregnant women without overweight^#^ (BMI<25 kg/m^2^, N=9394) | 1.31 (1.05-1.63) | .015 |
| Pregnant women who are not drinkers (N=9916) | 1.18 (1.01, 1.38) ^c^ | .039 |
| Pregnant women without a family history of diabetes (N=6899) | 1.24 (1.03, 1.48) ^d^ | .021 |
| Pregnant women with non-smoking (N=9730) | 1.11 (.90, 1.37) ^e^ | .32 |
| Pregnant women without history of adverse pregnancy outcomes (N=7956) | 1.73 (1.26, 2.36) ^f^ | .001 |

*, Chinese standard for overweight; #, international standard for overweight.

a, adjusted for pre-BMI, gestation weeks, education, gravidity, history of adverse pregnancy outcomes, family history of diabetes, folic acid supplementation, smoking and drinking status;

b, adjusted for age, gestation weeks, education, gravidity, history of adverse pregnancy outcomes, family history of diabetes, folic acid supplementation, smoking and drinking status;

c, adjusted for age, pre-BMI, gestation weeks, education, gravidity, history of adverse pregnancy outcomes, family history of diabetes, folic acid supplementation, and smoking status;

d, adjusted for age, pre-BMI, gestation weeks, education, gravidity, history of adverse pregnancy outcomes, folic acid supplementation, smoking and drinking status;

e, adjusted for age, pre-BMI, gestation weeks, education, gravidity, history of adverse pregnancy outcomes, family history of diabetes, folic acid supplementation, and drinking status;

f, adjusted for age, pre-BMI, gestation weeks, education, gravidity, family history of diabetes, folic acid supplementation, smoking and drinking status;

SPL, spontaneous pregnancy loss; HbA1c, glycated hemoglobin A1c; RR, risk ratio.

# The Shanghai PreConception Cohort (SPCC) group

Guoying Huang, Weili Yan, Xiaojing Ma, Weifen Luo, Wei Sheng, Yi Zhang, Yuan Jiang, Yin Ye, Dingmei Wang, Xiaotian Chen, Mengru Li, Hongyan Chen, Mi Ji, Yumei Liu, Gu Qing(s), Gu Qing(o), Linmei Zhu, De’ai Hou, Peiyu Sun, Xupeng Sun (Children’s Hospital of Fudan University, Shanghai, China); Hongbing Wang, Li Meng, Lin Zhang (Jingan Maternal and Child Health Center, Shanghai, China) ; Zifen Dai, Li fen (Shanghai First Maternity and Infant health Hospital, Shanghai, China); Shufang Chen, Zhenhua Tang, Jiahao Wu (International Peace Maternal and Child Health Hospital, Shanghai, China); Shuhua Wang, Dan li, Hui Wang (Xuhui Maternal and Child Health Center, Shanghai, China); Yu Ke, Weiping Cao, Baoren Zhang, Hong Huang (Shanghai Pudong New Area Health Care Hospital for Women & Children, Shanghai, China); Nailing Wang, Min Jiang, Jie Chen, Qiumin Xia (Shanghai Punan Hospital of Pudong New District, Shanghai, China); Hui Xu, Guoying Lao (Changning Maternity and Infant Health Hospital, Shanghai, China); HongMei Jin, Wenjuan Xie, Pin Yi (Qingpu Hospital, Zhongshan Hospital, Shanghai, China); Weiming Gong, JianXin Xu, Yingying Qian (Shanghai Qingpu Maternal and Child Health Center, Shanghai, China); Mingjie Luo, Jingwei Xia, Dongmei Chen, Zhenyu Tang (Shanghai Huangpu Maternal and Child Health Center, Shanghai, China); Xuejing Zhu, Qing Liu, Huiling Yang (Shanghai Huangpu Maternal and Child Health Hospital, Shanghai, China); Xiaotian Li, Zhiyong Wu, Chuanmin Ying, Shan Shi (Obstetrics and Gynecology Hospital of Fudan University (Shanghai Red House Obstetrics and Gynecology Hospital, Shanghai, China); Yanquan Zhang, Mingyi Yang (Wujing Hospital, Minhang District, Shanghai, Shanghai, China); Xiaohua Zhang, Lei Zhang, Lin Guan (Shanghai Minhang District Maternal and Child Health Care Hospital, Shanghai, China); Jinyu Xu, Honglin Wang, Fang Shen (The Fifth People's Hospital of Shanghai, Fudan University, Shanghai, China); Wenying Li, Xiaojing Teng, Jinling Zhao (Shanghai Minhang TCM Hospital, Shanghai, China); Cuili Zhu, Lan Wang, Hongwei Chen (Shanghai Songjiang District Central Hospital, Shanghai, China); Xiaoming Yuan, Meihua Zhang, Yaqiong Jin (Sijing Hospital, Songjiang District, Shanghai, China); Qing Yang, Wu Yan, Ying Wang, Hong Zhu, Min Feng (Songjiang Maternal and Child Health Center, Shanghai, China); Ying Wang, Yan Wu, Hong Tang (Songjiang Maternal and Child Health Hospital, Shanghai, China); Sa Guo (Tongji Hospital of Tongji University, Shanghai, China); Hongling Du (Shanghai Putuo District People's Hospital, Shanghai, China); Yuhuan Liu, Zhanyue Yi, Renhua Shi (Changhai Hospital, Second Military Medical University, Shanghai, Shanghai, China); Yu Gu, Qinfen Su, Yingying Lv (Shanghai Zhabei District Central Hospital, Shanghai, China); Yun Sun, Qiongpei Gu (Yangpu District Family Planning Service Center, Shanghai, China); Xixia Pang, Qingwu Zhang (Kong Jiang Hospital of Yangpu District, Shanghai, China); Songxiao Bai, Baoqiao Qi (Shanghai East City Hospital, Shanghai, China);

Dong Junyin, Xie Rong, Gu Caihua, Zhang Xiangrong, He Hui, Shen Meifang, Tang Xiaxia, Zhang Shuanghu, Zhou Chengyan, Zhang Xiaoying, Yang Lili, Yao Hongping, Dai Sijia, Song Liujuan, Li Juan, Li Sibei, Wu Guirong, Gu Xiaowen, Gu Mingying, Qu Chunyan, Yan Bei, Wu Huijing, Tan Haiyun, Huang Ruonan, Shi Yongjie, Wang Zheng (Community Health Centers of Minhang District, Shanghai, China); Gu Xueyin, Zhou Ying, Zhu Weifang, Zou Hong, Lu Xinhua, Yao Huahui, Lin Xiangying, Zhang Hongwei, Wu Xiaofeng, Shen Ying, Ma Shengyan, Liang Xinxing, Wang Qing, Zhou Mingying, Ni Li, Ye Honglian, Li Jiayi, Gao Chunyan, Lu Xiuqin, Yin Qiuyi, Jin Jieping, Yan Lei, Meng Fanxin, Zhang Lan, Li Na, Chen Ru, Gu Zhufang, Tao Wenwei, Qiu Zhongfen, Gu Hongwei, Wu Weirong (Community Health Centers of Songjiang District, Shanghai, China).
